# Supplementary material for: Design of a unidirectional water valve in Tillandsia
Source: Nat Commun. 2020 Jan 20;11:396. doi: 10.1038/s41467-019-14236-5 (PMC6971078; doi:10.1038/s41467-019-14236-5)
Supplement: Supplementary file 1 — Supplementary Information [file 41467_2019_14236_MOESM1_ESM.pdf]

# Supplementary Information

# Design of a Unidirectional Water Valve in *Tillandsia*

**Pascal S. Raux, Simon Gravelle, Jacques Dumais**

**Universidad Adolfo Ibáñez**
**Facultad de Ingeniería y Ciencias**
**Viña del Mar, Chile**

**jacques.dumais@uai.cl**

- Supplementary Figs. 1-10
- Supplementary Note

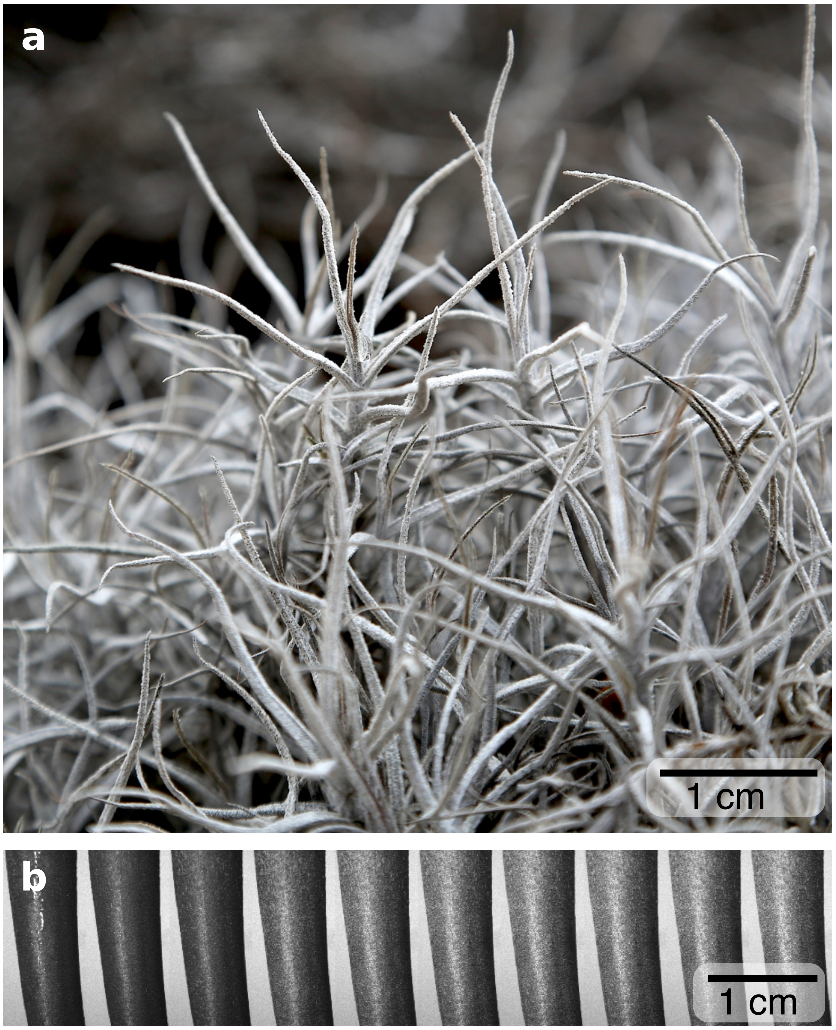


**Supplementary Figure 1**: **Trichome layer in *Tillandsia***. (**a**) Close-up of the leaves of *T. landbeckii*. The white powdery aspect of the leaves reflects the dense layer of hydrophilic trichomes. (**b**) Drying of the thin water film on the surface of *T. aeranthos* following exposure to fog (the same leaf shown at 5 min. intervals). As the surface water evaporates, the leaf recovers its lighter aspect characteristic of dry trichomes. The surface water plays the role of a water reservoir for evaporation. All our measurements were done after this first phase of evaporation to ensure we were measuring the intrinsic resistance of the leaf.

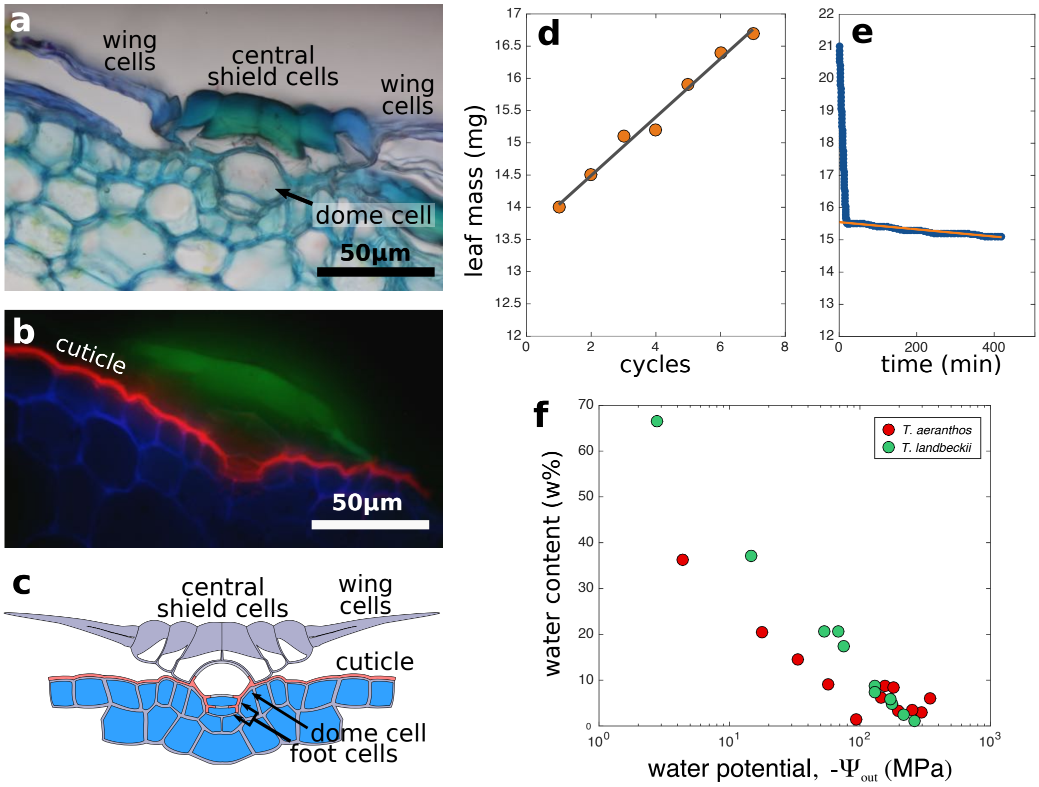


**Supplementary Figure 2: Structure and function of the *T. landbeckii* trichome.** (**a**) Transmitted light micrograph of a thin section of the trichome stained with toluidine blue. Note the thick wall of the central shield cells. (**b**) Composite fluorescence image of the trichome. The cell walls of the central shield cells and epidermis are highlighted by their own autofluorescence (green and blue) while the cuticle is marked with Sudan 3 (red). (**c**) Diagram of the trichome structure. Living cells are shown in blue while dead cells have their lumen shown in white. (**d**) Mass of a cluster of leaves after repeated immersions in water. From these experiments, we calculated an absorption rate of approximately $Q_{\mathrm{abs}} = +90\,\mathrm{mg\,m^{-2}\,min^{-1}}$. (**e**) Typical evaporation curve. A short phase of evaporation of the free surface water, the leaves reach their characteristic evaporation rate. From these experiments, we calculated an evaporation rate of approximately $Q_{\mathrm{eva}} = -2.6\,\mathrm{mg\,m^{-2}\,min^{-1}}$ (**f**) Water content of excised shield walls as a function of the relative humidity.

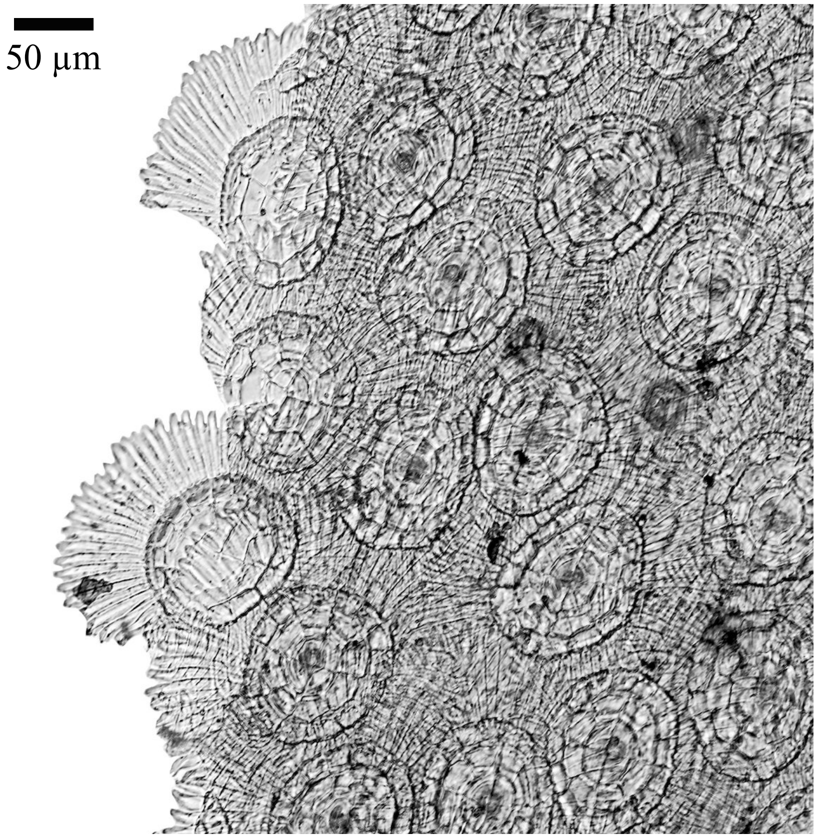


**Supplementary Figure 3: Transmitted light micrograph of a paradermal section of a *T. aeranthos* leaf showing the extensive overlap of the trichome wings.**

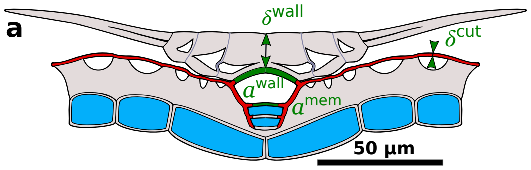


**b**

| Parameters | Values |
|---|---|
| Shield wall thickness | $\delta^{wall}$ = 21.3 ± 3.6 µm |
| Cuticle thickness | $\delta^{cut}$ = 1.8 ± 0.4 µm |
| Dome cell outer wall area | $a^{wall}$ = 650 ± 30 µm$^2$ |
| Foot cell membrane area | $a^{mem}$ = 150 ± 10 µm$^2$ |
| Trichome density | $n^{tri}$ = 1.75 · 10$^8$ m$^{-2}$ |

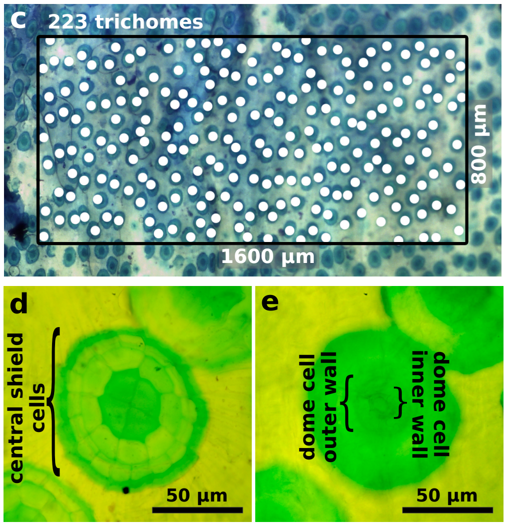


**Supplementary Figure 4: Key parameters for the trichome.** (**a**) Trichome diagram indicating the key geometrical parameters: the cuticle thickness $\delta^{cut}$, the thickness of the outer shield wall $\delta^{wall}$, the surface area of the outer wall of the dome cell $a^{wall}$, and the surface area of the plasma membrane of the outermost foot cell $a^{mem}$. (**b**) Geometrical parameters for the *T. aeranthos* trichome (mean ± std, $n = 5$). (**c**) Example of trichome surface density measurement, each white dot is a detected trichome. (**d**) Micrographs of the *T. aeranthos* trichome at the level of the outer shield walls. The area of the central shield cells is 6500 µm$^2$. (**e**) Micrographs of the *T. aeranthos* trichome at the level of the dome cell. Note the two concentric circles representing the outer wall and inner wall of the dome cell (compare with $a^{wall}$ and $a^{mem}$ in (**b**)). The sections in (**d**) and (**e**) were stained with toluidine blue.

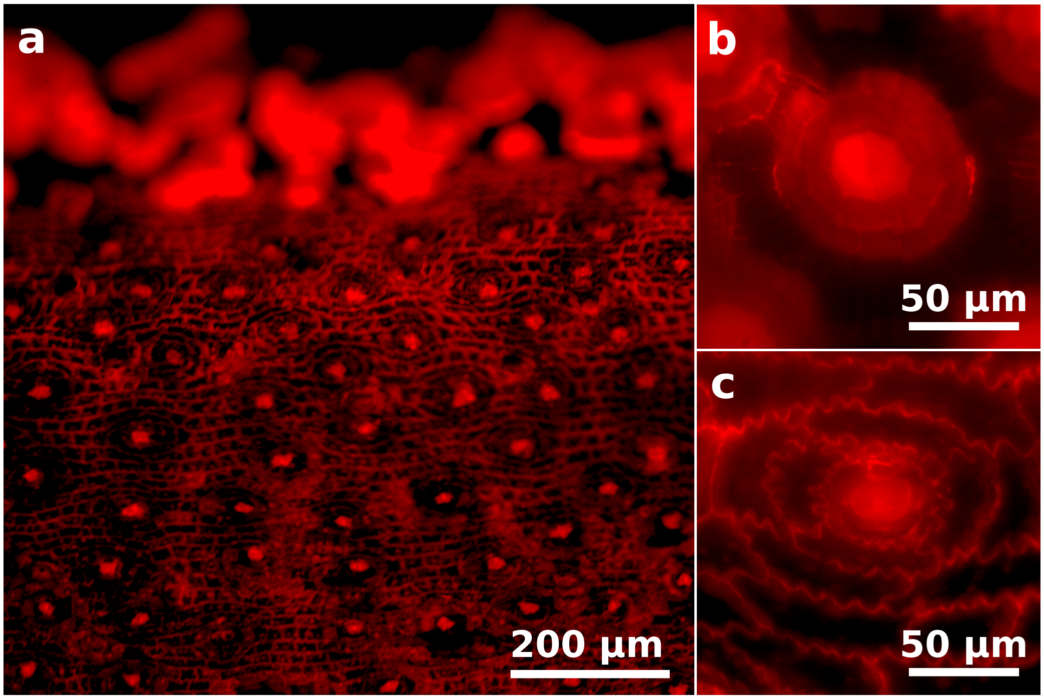


**Supplementary Figure 5: Shaving experiments.** (**a**) Fluorescence micrograph of the *T. aeranthos* after shaving the trichomes with a razor blade. The walls are stained with propidium iodide. Note the trichomes shield are still present in the upper tier of the picture while only the "sockets" remain after shaving in the lower tiers of the picture. (**b**) Close-up of an intact trichome. (**c**). Close-up of the socket remaining after shaving.

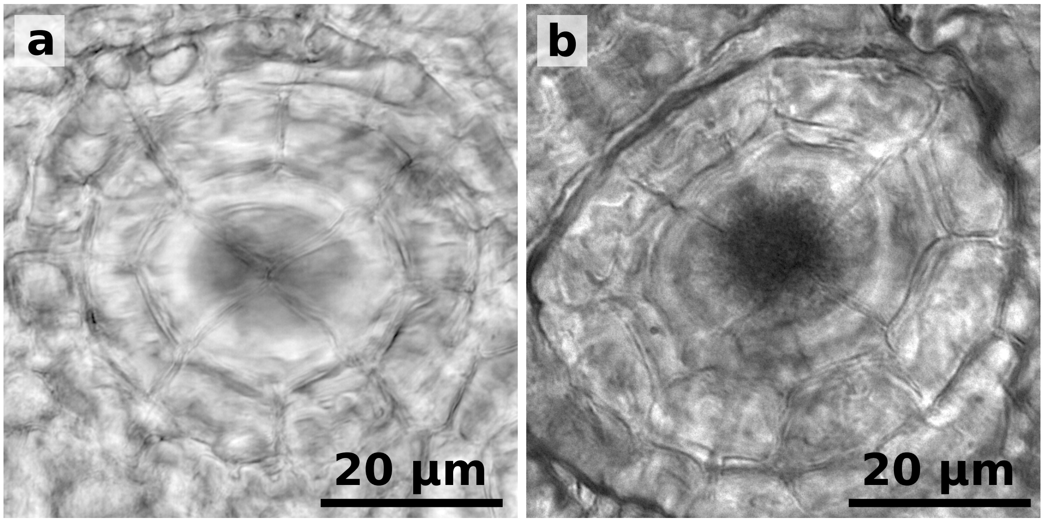


**Supplementary Figure 6: Solute accumulation in *T. aeranthos*.** (**a**) Transmitted light micrograph of an untreated trichome. (**b**) Trichome exposed for several hours to $CuSO_4$. Note the dark mass indicating the accumulation of $CuSO_4$ in the center of the trichome.

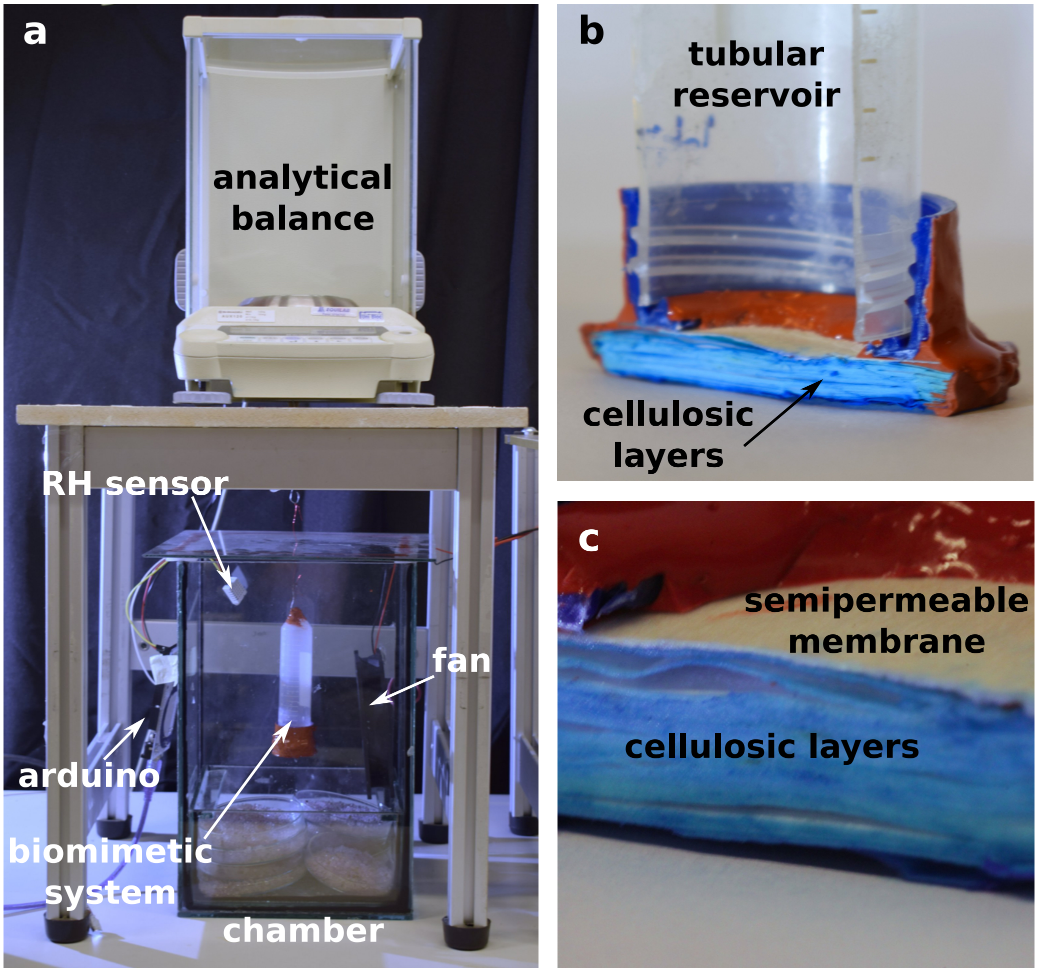


Supplementary Figure 7: Experimental set-up.

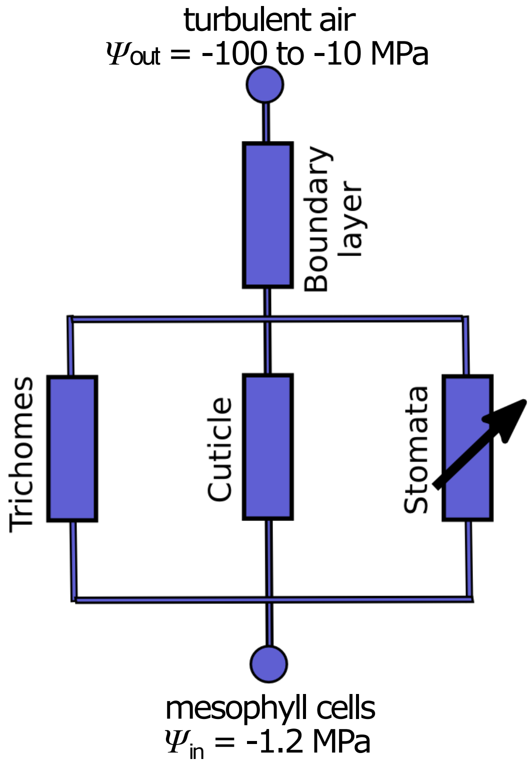


**Supplementary Figure 8: The possible paths of water evaporation in *Tillandsia* leaves.**

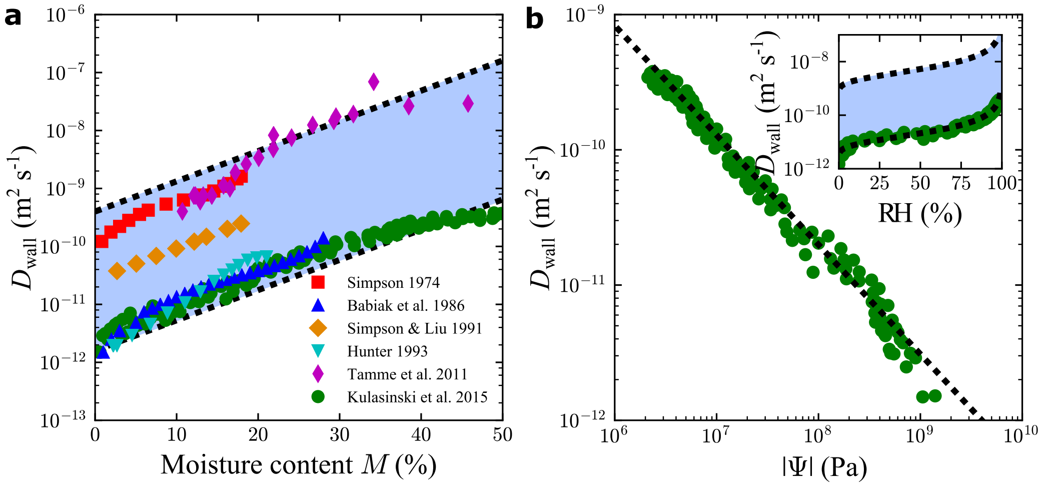


**Supplementary Figure 9: Influence of the water potential on the transport properties of cellulose.** (**a**) Effective water diffusion coefficient in cellulose $D_{\mathrm{cell}}$ function of moisture content $M$ for various woods[27;28;29;30;31] as well as from molecular dynamics simulations in cellulose[32] (green circles). In the range relevant for our study ($M < 40\%$), the data sets share a common trend despite the variation of methods (and pore microstructures): in all cases the diffusion coefficient decreases as the moisture content is reduced (i.e. as $|\Psi|$ increases), following an empirical law $A\exp(\alpha M)$ where the coefficients for the lower (resp. upper) fits are $\alpha \approx 0.12\ \%^{-1}$ and $A = 1.6 \cdot 10^{-12}\ \mathrm{m^2\,s^{-1}}$ (resp. $4 \cdot 10^{-10}\ \mathrm{m^2\,s^{-1}}$) (domain in light blue, both limits are shown as dash lines). (**b**) Effective water diffusion coefficient in cellulose $D_{\mathrm{cell}}$ from molecular dynamics simulations[32] showing the variation with the water potential $|\Psi| = -\Psi$. The dash line is a fit $D_{\mathrm{cell}} \sim 6.0 \cdot 10^{-5}|\Psi|^{-0.8}$. The inset presents the same data function of the relative humidity, showing in light blue the domain corresponding to the same span than in the previous fits of panel (**a**).

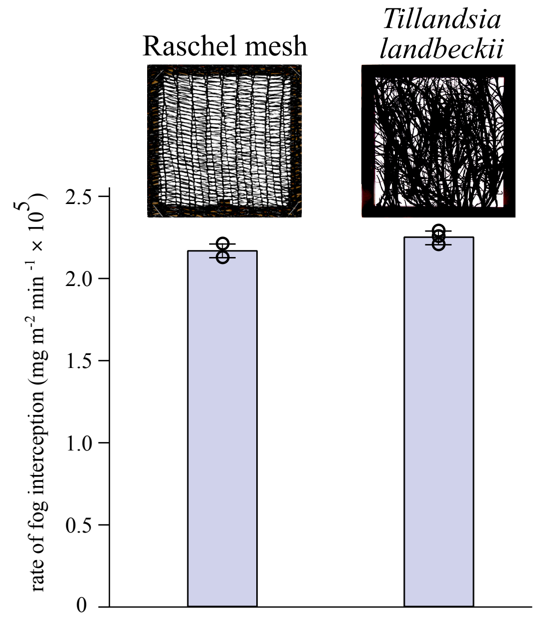


**Supplementary Figure 10: The rate of water capture for the traditional Raschel mesh of fog collectors and an equivalent cluster of *Tillandsia landbeckii* leaves.**

## Supplementary Note

This Supplementary Note presents the quantitative analyses necessary to infer the conductances associated with the different elements of the *Tillandsia* trichome.

**Water potential, flux and conductance.** The water potential $\Psi$ is defined relative to a reference potential $\Psi_0$ which customarily is taken as the potential of pure water at standard temperature and pressure. Relative to this reference potential, the water potential of plant cells is typically negative and has two principal contributions. First, there is an entropic contribution from dissolved substances that gives rise to the osmotic potential $\Psi_\Pi = RT\ln(\gamma n)/V_w$ where $R$ the gas contant, $T$ the temperature, $V_w$ the molar volume of water, $\gamma$ the dimensionless activity coefficient and $n$ the water mole fraction[33]. The water potential of the cells also includes a hydrostatic potential $\Psi_\mathrm{P}$ corresponding to the hydrostatic pressure that arises from the elastic deformation of the cell walls and potentially the Laplace pressure associated with the numerous air-water interfaces in the interstices of cell walls. All together, the potential inside the cells of plants is given by the relation[34]:

$$\Psi_\mathrm{in} = \Psi_\Pi + \Psi_\mathrm{P} + \Psi_0, \tag{1}$$

and typically ranges from to $-1$ to $-0.1$ MPa. Note that gravitational contributions to the water potential have been neglected. On the other hand, the water potential in the environment outside the leaf $\Psi_\mathrm{out}$ depends on the temperature and relative humidity (RH) of the air:

$$\Psi_\mathrm{out} = \frac{RT}{V_w}\ln(\mathrm{RH}) + \Psi_0. \tag{2}$$

where $R = 8.314 \cdot 10^{-6}\,\mathrm{m^3\,MPa\,K^{-1}\,mol^{-1}}$ and $V_w = 18 \cdot 10^{-6}\ \mathrm{m^3\,mol^{-1}}$. For $T = 293°\mathrm{K}$ and a relative humidity of RH = 50%, the water potential of the air $\Psi_\mathrm{out}$ is equal to $-94\,\mathrm{MPa}$. For RH = 20%, $\Psi_\mathrm{out}$ is as negative as $-218\,\mathrm{MPa}$. In contrast, air saturated with fog has a water potential of $\Psi_\mathrm{out} \approx \Psi_0 = 0\,\mathrm{MPa}$.

When the water potential inside the cells of a plant ($\Psi_\mathrm{in}$) differs from the outside potential ($\Psi_\mathrm{out}$), a net water movement is expected from the region of high water potential to the region of low water potential. Under

the assumption that the system is close to equilibrium, stationary and one-dimensional, the mass flux density of water $Q$ ($\mathrm{g\,m^{-2}\,s^{-1}}$) is proportional to the difference in water potential, $\Delta\Psi = \Psi_{\text{out}} - \Psi_{\text{in}}$ (MPa), across the membrane restricting the flow:

$$Q = \mathcal{L}\ \Delta\Psi. \tag{3}$$

The proportionality factor $\mathcal{L}$ ($\mathrm{g\,m^{-2}\,s^{-1}\,MPa^{-1}}$) is the conductance of the membrane to water flow (and is the inverse of the resistance $\mathcal{R}$). It is sometimes more convenient to use the difference in water concentration $\Delta c$ ($\mathrm{mol\,m^{-3}}$) as the thermodynamic "force" driving flow instead of the difference in water potential. This is the case when dealing with the vapor flux arising during transpiration where the one-dimensional first Fick's law yields:

$$Q = M_w D \frac{\Delta c}{\delta}, \tag{4}$$

where $D$ ($\mathrm{m^2\,s^{-1}}$) is the water diffusion coefficient in the medium under consideration, $\delta$ is the distance over which diffusion takes place, and $M_w = 18\,\mathrm{g\,mol^{-1}}$ is the molar mass of water.

In case of a flux of vapor, equations (3) and (4) are equivalent, and one can identify the conductance of the membrane:

$$\mathcal{L} = \langle c \rangle \frac{V_w}{RT} \frac{M_w D}{\delta}, \tag{5}$$

where we used $\Delta\Psi \approx \frac{RT}{V_w} \frac{\Delta c}{\langle c \rangle}$ following $\Psi = \frac{RT}{V_w} \ln(\mathrm{RH})$. Note that $\langle c \rangle$ is the average concentration within the medium, that can be chosen as $\langle c \rangle = (c_{\text{ext}} + c_{\text{in}})/2$.

**Liquid water absorption.** The liquid water present on the surface of a *Tillandsia* leaf must cross successively the thick outer walls of the trichome and the plasma membrane of the foot cells before it can reach the living mesophyll. Our experiments, however, already indicate that the shield walls offer little resistance to the inner flow of liquid water. Specifically, we have shown that shaved leaves only increase their rate of water absorption by 12.5 % as compared to intact leaves (Fig. 4d).This increase is not statistically significant as determined by a Student t-test ($P > 0.2$). The same conclusion is reached from our experiments with $CaCl_2$ solutions

(Fig. 3e). Since it is known that *Tillandsia* species acquire minerals and other nutrients through their trichomes[35], we expect the osmotic solution used in these experiments to permeate the entire outer shield and come in direct contact with the plasma membrane of the outermost foot cell. The resistance to the outward flow of liquid water observed in these treatments is therefore imputable to the plasma membrane and indicate that the resistance measured for the inward flow of fog water by the leaf is the same as the resistance to the outward flow under an osmotic grandient (Fig. 3e). We conclude that the rate of inward or outward flow of *liquid* water is set by the permeability of the plasma membrane of the foot cells. Given this conclusion, the plasma membrane resistance can be calculated as $\mathcal{R}^{\mathrm{mem}} = (n^{\mathrm{tri}} a^{\mathrm{mem}}) \Delta\Psi_{\mathrm{abs}} / Q_{\mathrm{abs}}$, where $Q_{\mathrm{abs}} = (240 \pm 60)\,\mathrm{mg\,min^{-1}\,m^{-2}}$ is the water absorption rate measured on intact leaves, $\Delta\Psi_{\mathrm{abs}} = 1.2\,\mathrm{MPa}$ is the water potential difference, $n^{\mathrm{tri}} = 1.75 \cdot 10^{8}\,\mathrm{m^{-2}}$ is the trichome area density and $a^{\mathrm{mem}} = (1.5 \pm 0.1) \cdot 10^{-10}\,\mathrm{m^2}$ is the area of the plasma membrane of the foot cell (Supplementary Fig. 4). The prefactor $n^{\mathrm{tri}} a^{\mathrm{mem}} = 0.026$ is a geometrical correction accounting for the fact that the total area of the foot cells' membranes accounts for only a small fraction of the total leaf area. Based on this equation, the membrane resistance is $\mathcal{R}^{\mathrm{mem}} = (1.3 \pm 0.3) \cdot 10^{-4}\,\mathrm{MPa\,min\,m^2\,mg^{-1}}$ which is in good agreement with the range of resistance reported for other plant membranes[36].

**Water evaporation.** Unlike absorption of liquid water, the paths for evaporation are potentially numerous and complex (Supplementary Fig. 8). Since we are interested in the specific transport properties of the trichome, we must evaluate the impact of other evaporation paths that may be acting in parallel with the trichome path. Two main parallel paths exist. First, a substantial fraction of the leaf water could be lost by stomatal transpiration. Schmitt and coworkers working with *T. recurvata* estimated that the conductance of the stomata on a per leaf basis is approximately six times the conductance of the trichomes[37]. However, *Tillandsia* species are CAM (Crassulacean Acid Metabolism) plants and, as such, respiration and the associated stomatal transpiration are observed only at night[37]. We also observed some evidence of diurnal fluctuations in the rate of evaporation (Fig. 2a, inset) but have not attempted to repeat the experiments of Schmitt *et al.* since all of our experimental treatments were done during the day and showed highly significant effects on the rate of evaporation without having to distinguish between the stomatal and trichome transpiration (Fig. 4).

Nonetheless, the reader should keep in mind that the evaporative fluxes reported for the intact leaves are an upper bound for the actual evaporation of the trichomes. Considering that the plants were respiring for 12 hours daily, the actual evaporative loss at the level of the trichomes could be four times lower than the value we reported.

The second path for evaporation is the leaf cuticle which is also slightly permeable to water (Supplementary Fig. 8). However, due to the relatively low diffusion coefficient of water in wax $D_{\text{wax, w}} = 5 \cdot 10^{-14}\,\text{m}^2\text{s}^{-1}$[38], and using a cuticle thickness of $\delta^{\text{cut}} = (1.8 \pm 0.4)\,\mu\text{m}$ (Supplementary Fig. 4), the resistance of the cuticle is expected to be about 30 times greater than the resistance of the trichomes, which confirms its minor contribution to the total evaporative losses.

A boundary layer resistance is present in all of our experimental treatments (Supplementary Fig. 8), although we have reduced its contribution to a minimum by maintaining air circulation around the leaf with the help of a fan. Using an estimated boundary layer thickness of $\delta^{\text{bdr}} = 1\,\text{mm}$, one gets $\mathcal{R}^{\text{bdr}} = (8.1 \pm 4.3) \cdot 10^{-3}\,\text{MPa}\,\text{min}\,\text{m}^2\,\text{mg}^{-1}$[34]. Thus, the boundary layer is expected to contribute less than 0.005% to the overall resistance. We can test this conclusion by measuring the evaporation rate of the free surface water on the leaf since the latter is controlled only by the boundary layer. We found an evaporation rate for the free surface water 360 times higher than the basal rate of evaporation for the leaf (Fig. 4d), thus confirming that the boundary layer is not the main factor controlling the rate of evaporative losses.

Given the considerations stated above, the resistance of the trichome shield can be estimated from the measured overall evaporative flux as $\mathcal{R}^{\text{wall}} = (n^{\text{tri}}a^{\text{wall}})\Delta\Psi_{\text{eva}}/Q_{\text{eva}} = (3.3 \pm 2.0)\,\text{MPa}\,\text{min}\,\text{m}^2\,\text{mg}^{-1}$, where $Q_{\text{eva}} = -(3.2 \pm 2.0)\,\text{mg}\,\text{min}^{-1}\,\text{m}^{-2}$, $\Delta\Psi_{\text{eva}} = -94$ MPa $-(-1.2$ MPa$) = -92.8$ MPa, and, as before, a geometrical factor $n^{\text{tri}}a^{\text{wall}} = (1.75 \cdot 10^8\,\text{m}^{-2}) \cdot (6.5 \cdot 10^{-10}\,\text{m}^2)$ is used to infer the intrinsic resistance of the trichome. From eq. (5), we deduce an effective diffusion coefficient for water vapor inside the trichome cellulosic wall equal to $D_{\text{wall}} = (1.1 \pm 0.8) \cdot 10^{-9}\,\text{m}^2\ \text{s}^{-1}$, where we used $\langle c \rangle = 0.72\,\text{mol}\,\text{m}^{-3}$ and a wall thickness of $\delta^{\text{wall}} = (21.3 \pm 3.6)\,\mu\text{m}$. The measured diffusion coefficient (at RH $\approx$ 50%) is in good agreement with values published elsewhere in the literature[39;32]. Note that in series with

the cellulose shield, the cell membrane is expected to contribute to less than 0.002% to the overall resistance of the trichome if we refer to the value of the cell membrane extracted in the "Liquid water absorption" subsection, $\mathcal{R}^{\text{mem}} = (1.3 \pm 0.3) \cdot 10^{-4}\,\text{MPa}\,\text{min}\,\text{m}^2\,\text{mg}^{-1}$.

In the discussion above, we considered the properties of cellulose at a fixed relative humidity RH = 50%. While eq. (3) is still valid, the value of the conductance $\mathcal{L}$ depends on the relative humidity: the effective diffusion coefficient of water in cellulosic materials $D_{\text{wall}}$ varies strongly with the water potential, as shown by the extensive literature on wood drying[40;31;27;28;29;30;41]. However, due to the diversity of cellulose and wood microstructures, measurements do not fall on a unique universal law for $D_{\text{wall}}$(Supplementary Fig. 9a) [31;27;28;29;30;41]. Despite this diversity, these results share a common trend: the decrease of the diffusion coefficient ($D_{\text{wall}}$) with decreasing moisture content ($M$) can be fitted by an exponential although the prefactor can vary by a factor 250 (blue domain in Supplementary Fig. 9a). Results from molecular dynamics simulations[32] lead to a similar relationship between $D_{\text{wall}}$ and $M$, at least for moderate water content ($M < 40\%$).

In order to quantify the effect of water content, we use the latter data set on which the diffusion coefficient was measured simultaneously with the moisture content and the water potential[32]. Supplementary Fig. 9b shows that these measurements are in good agreement with a law $D_{\text{wall}} \approx 6.0 \cdot 10^{-5}|\Psi|^{-0.8}$. From the comparison with measurements on wood, this law is a lower bound for the diffusion coefficient, and an upper bound is expected by multiplying the prefactor by 250, i.e. $D_{\text{wall}} \approx 1.5 \cdot 10^{-2}|\Psi|^{-0.8}$ (blue domain in Supplementary Fig. 9b). Finally, from this empirical law for $D_{\text{wall}}(\Psi)$, we are able to estimate the expected conductance of cellulosic material from eq. (5), using the same geometrical considerations developed above:

$$\mathcal{L}_{\text{wall}} \sim \frac{\langle c \rangle\, V_w M_w D_{\text{wall}}(\Psi)}{RT\delta^{\text{wall}}} n^{\text{tri}} a^{wall} \tag{6}$$

with $\delta^{\text{wall}}$ is the thickness of the shield wall. Using $|\Psi| \approx -\frac{RT}{V_w}\ln \text{RH}$ and $\langle c \rangle \approx \text{RH}\frac{p_{\text{sat}}}{2RT}$ ($p_{\text{sat}} \approx$ 2.34 kPa), this equation yields the domain displayed in the inset of Fig. 3f.

**Comparison with biomimetic composite membranes.** We repeated our absorption and evaporation experiments with a simple biomimetic sys-

tem inspired by the *Tillandsia* trichome. The system is made of an osmotic NaCl solution ($\Psi_{in} = -4.5\,\text{MPa}$) separated from the environment by a semipermeable membrane and a cellulosic layer. For absorption of water through the semipermeable membrane only, a mass flow of $Q_{\text{abs}} = (2.3 \pm 0.4) \cdot 10^3\,\text{mg}\,\text{min}^{-1}\,\text{m}^{-2}$ was measured, and since $\Delta\Psi_{\text{abs}} = 4.5\,\text{MPa}$, the resistance to liquid water absorption is $\mathcal{R}_{\text{abs}} = \Delta\Psi_{\text{abs}}/Q_{\text{abs}} = (2.0 \pm 0.3) \cdot 10^{-3}\,\text{m}^2\,\text{MPa}\,\text{min}\,\text{mg}^{-1}$. In evaporation experiments, a mass flow of $Q_{\text{eva}} = -(1.1 \pm 0.2) \cdot 10^4\,\text{mg}\,\text{min}^{-1}\,\text{m}^{-2}$ was recorded, and since $\Delta\Psi_{\text{eva}} = -94$ MPa $-(-4.5$ MPa$) = -89.5\,\text{MPa}$, $\mathcal{R}_{\text{eva}} = (8.1 \pm 1.5) \cdot 10^{-3}\,\text{m}^2\,\text{MPa}\,\text{min}\,\text{mg}^{-1}$. From the two resistance values, we calculate an asymmetry ratio for the semipermeable membrane alone of $\mathcal{R}_{\text{eva}}/\mathcal{R}_{\text{abs}} = 4.1$. In other words, a system equipped with only a semipermeable membrane shows very little transport asymmetry under normal conditions.

In a second series of experiments, we worked with composite structures made of a semipermeable membrane and a layer of cellulose. The cellulose layer was made of $N$ (up to 100) sheets of printer paper with density $\rho_s = 75\,\text{g/m}^2$, each sheet with a thickness of $100\,\mu\text{m}$. For these experiments, $\mathcal{R}_{\text{abs}} = (1.8 \pm 0.3) \cdot 10^{-3}\,\text{m}^2\,\text{MPa}\,\text{min}\,\text{mg}^{-1}$ and $\mathcal{R}_{\text{eva}} = (9.5 \pm 1.8) \cdot 10^{-1}\,\text{m}^2\,\text{MPa}\,\text{min}\,\text{mg}^{-1}$, leading to an asymmetry ratio of $\mathcal{R}_{\text{eva}}/\mathcal{R}_{\text{abs}} = 530$, which is more than 100 times the asymmetry of the naked semipermeable membrane. Although this asymmetry remains modest compared to the *Tillandsia* trichome, it is likely that the asymmetry could be improved by using a more conductive semipermeable membrane and optimizing the layer of cellulose to maximize its resistance to water vapor diffusion.

## Supplementary References


[27] Babiak, M., Pavlíková, M. & Jokel, J. Modelling of the transport of water in wood. *IFAC Proceedings Volumes* **19**, 139–144 (1986).

[28] Simpson, W. & Liu, J. Dependence of the water vapor diffusion coefficient of aspen (populus spec.) on moisture content. *Wood Science and Technology* **26**, 9–21 (1991).

[29] Hunter, A. On movement of water through wood - the diffusion coefficient. *Wood science and technology* **27**, 401–408 (1993).

[30] Tamme, V., Muiste, P., Mitt, R. & Tamme, H. Determination of effective diffusion coefficient and mechanical stress of pine wood during convective drying. *Baltic Forestry* **17**, 32 (2011).

[31] Simpson, W. T. Measuring dependence of diffusion coefficient of wood on moisture concentration by adsorption experiments. *Wood and Fiber Science* **5**, 299–307 (1974).

[32] Kulasinski, K., Guyer, R., Derome, D. & Carmeliet, J. Water diffusion in amorphous hydrophilic systems: A stop and go process. *Langmuir* **31**, 10843–10849 (2015).

[33] Eijkel, J. C. T. & van den Berg, A. Water in micro- and nanofluidics systems described using the water potential. *Lab on a chip* **5**, 1202–1209 (2005).

[34] Nobel, P. S. *Physicochemical and environmental plant physiology* (Academic press, 1999).

[35] Benzing, D. H. *Bromeliaceae: profile of an adaptive radiation* (Cambridge University Press, 2000).

[36] Maurel, C. Aquaporins and water permeability of plant membranes. *Annual Review of Plant Physiology and Plant Molecular Biology* **48**, 399–429 (1997).

[37] Schmitt, D.-B. A., Martin, C. & Lüttge, U. Gas exchange and water vapor uptake in the atmospheric cam bromeliad *Tillandsia recurvata* L.: the influence of trichomes. *Botanica Acta* **102**, 80–84 (1989).

[38] Veraverbeke, E. A., Verboven, P., Scheerlinck, N., Hoang, M. L. & Nicolaï, B. M. Determination of the diffusion coefficient of tissue, cuticle, cutin and wax of apple. *Journal of Food Engineering* **58**, 285–294 (2003).

[39] Topgaard, D. & Söderman, O. Diffusion of water absorbed in cellulose fibers studied with H-NMR. *Langmuir* **17**, 2694–2702 (2001).

[40] Tremblay, C., Cloutier, A. & Fortin, Y. Determination of the effective water conductivity of red pine sapwood. *Wood Science and Technology* **34**, 109–124 (2000).

[41] Perkowski, Z., Świrska-Perkowska, J. & Gajda, M. Comparison of moisture diffusion coefficients for pine, oak and linden wood. *Journal of Building Physics* **41**, 135–161 (2017).
