# Supplementary material for: Design of a unidirectional water valve in Tillandsia
Source: Nat Commun. 2020 Jan 20;11:396. doi: 10.1038/s41467-019-14236-5 (PMC6971078; doi:10.1038/s41467-019-14236-5)
Supplement: Supplementary file 3 — Reporting Summary [file 41467_2019_14236_MOESM3_ESM.pdf]

nature research

Corresponding author(s): Jacques Dumais

Last updated by author(s): Dec 3, 2019

Reporting Summary

Nature Research wishes to improve the reproducibility of the work that we publish. This form provides structure for consistency and transparency in reporting. For further information on Nature Research policies, see Authors & Referees and the Editorial Policy Checklist .

Statistics

For all statistical analyses, confirm that the following items are present in the figure legend, table legend, main text, or Methods section.

n/a	Confirmed	
☐	☒	The exact sample size (*n*) for each experimental group/condition, given as a discrete number and unit of measurement
☐	☒	A statement on whether measurements were taken from distinct samples or whether the same sample was measured repeatedly
☒	☐	The statistical test(s) used AND whether they are one- or two-sided<br>*Only common tests should be described solely by name; describe more complex techniques in the Methods section.*
☒	☐	A description of all covariates tested
☒	☐	A description of any assumptions or corrections, such as tests of normality and adjustment for multiple comparisons
☐	☒	A full description of the statistical parameters including central tendency (e.g. means) or other basic estimates (e.g. regression coefficient) AND variation (e.g. standard deviation) or associated estimates of uncertainty (e.g. confidence intervals)
☒	☐	For null hypothesis testing, the test statistic (e.g. *F*, *t*, *r*) with confidence intervals, effect sizes, degrees of freedom and *P* value noted<br>*Give P values as exact values whenever suitable.*
☒	☐	For Bayesian analysis, information on the choice of priors and Markov chain Monte Carlo settings
☒	☐	For hierarchical and complex designs, identification of the appropriate level for tests and full reporting of outcomes
☒	☐	Estimates of effect sizes (e.g. Cohen's *d*, Pearson's *r*), indicating how they were calculated

Our web collection on statistics for biologists contains articles on many of the points above.

Software and code

Policy information about availability of computer code

Data collection	Weight measurements: Shimadzu AUX120 balance interfaced with the computer using WindowsDirect<br>Pictures: Canon D200 camera and subsequent treatment with ImageJ 1.50i<br>Temperature and relative humidity measurements: Arduino equipped with a DHT22 sensor
Data analysis	Data analysis was performed with Matlab 2018a

For manuscripts utilizing custom algorithms or software that are central to the research but not yet described in published literature, software must be made available to editors/reviewers. We strongly encourage code deposition in a community repository (e.g. GitHub). See the Nature Research guidelines for submitting code & software for further information.

Data

Policy information about availability of data

All manuscripts must include a data availability statement. This statement should provide the following information, where applicable:

- Accession codes, unique identifiers, or web links for publicly available datasets
- A list of figures that have associated raw data
- A description of any restrictions on data availability

The data that support the findings of this study are available from the corresponding author upon request.

Field-specific reporting

Please select the one below that is the best fit for your research. If you are not sure, read the appropriate sections before making your selection.

Life sciences study design

All studies must disclose on these points even when the disclosure is negative.

Sample size	We attempted to have all of our key measurements repeated on at least five leaves, although this number was not based on any specific statistical model. The sample sizes for the quantitative data are:<br>Fig. 2a: one leaf shown as illustration but measurements of absorption and evaporation were repeated on n=8 leaves,<br>Fig. 2b: n=6,<br>Fig. 3e: n=5,<br>Fig. 3f: n=4,<br>Fig. 4a-c: one leaf shown as illustration,<br>Fig. 4d: n as listed in the table,<br>Fig. 5b: results for one biomimetic system<br>Fig. 5c: 5 different systems each averaged over more than 12 hours.<br>Fig. 5d: 1 system exposed to different RH with each measurement averaged over more than 12 hours.
Data exclusions	No data were excluded from the analyses once a robust protocol was established. For example, initial evaporation measurements (Fig. 2a and b) were made without a fan in the evaporation chamber. These measurements were excluded once it was found that the boundary layer of the leaves was affecting the measurements. Once this improvement to the experimental protocol was made, all further measurements were kept for the analysis.
Replication	All experimental results were replicated multiple times and, in most cases, by two investigators at two different times. The initial results (Fig. 2a, and Fig. 4) were first obtained by a laboratory technician (Aymeric Pinel, see acknowledgement). All of these experiments were later repeated more systematically by Pascal Raux because of concerns about the effect of the boundary layer on the results (see data exclusions). The new measurements varied slightly from the earlier ones but the conclusions were the same.
Randomization	Randomization was not used in this study. The most important results (Fig. 2b; Fig. 3e,f; Fig. 4, Fig. 5) involved measuring the response of the Tillandsia leaves and our biomimetic system to finely graded environmental parameters such as relative humidity and osmotic potential. Since clear trends emerged from all of these experiments, a randomized protocol was deemed unnecessary. However, the sequence in which the different external conditions were applied was somewhat randomized.
Blinding	Blinding was not used in this study because the response of the system did not involve any subjective evaluation from the researcher. All measurements were recorded directly by a computer and did not involve any "judgement call" from the researcher.

Reporting for specific materials, systems and methods

We require information from authors about some types of materials, experimental systems and methods used in many studies. Here, indicate whether each material, system or method listed is relevant to your study. If you are not sure if a list item applies to your research, read the appropriate section before selecting a response.

Materials & experimental systems

n/a	Involved in the study	
☒	☐	Antibodies
☒	☐	Eukaryotic cell lines
☒	☐	Palaeontology
☒	☐	Animals and other organisms
☒	☐	Human research participants
☒	☐	Clinical data

Methods

n/a	Involved in the study	
☒	☐	ChIP-seq
☒	☐	Flow cytometry
☒	☐	MRI-based neuroimaging
